# Prediction and Planning of Sports Competition Based on Deep Neural Network

**DOI:** 10.1155/2022/1906580

**Published:** 2022-06-08

**Authors:** Jin Xu

**Affiliations:** School of Sports Hunan City University, Yiyang 413000, China

## Abstract

Physical education curriculum has been paid more and more attention by teachers and parents, and having a healthy body is the foundation. School sports competition is also more and more concerned by major researchers, and scholars have produced in-depth research and analysis of sports competition results prediction because prediction results can better let teachers carry out appropriate sports training for students, so as to achieve the best learning effect. The construction of the prediction model and whether the performance and universality of the model after construction are suitable for predicting sports competitions have also become a major research point. Deep neural network is a complex network method to analyze the structure of the human brain, which plays a core role in the field of sports planning and performance prediction and can know the future performance of athletes or students in advance in sports competitions. This paper establishes the autoregressive summation model prediction model, the complex neural network prediction model, and the improved complex neural network prediction model. It is concluded that only the improved BP neural network model has a remarkable effect on performance prediction, and the prediction value obtained by this prediction model can reduce the systematic error of prediction, so that it can better infer the performance prediction of sports competitions in China and plan which sports events are suitable for which prediction model.

## 1. Introduction

We live in the education by leaps and bounds in the environment, and our physical education curriculum content and structure of the construction is becoming more and more complete and having rapid development. Based on humidity, dew point, wind speed, and other meteorological parameters, a depth neural network (DNN) model is proposed to predict the minimum and maximum temperature. A particle swarm optimization algorithm is applied to select the correlation and important features of the data set to improve the prediction accuracy of the model. In the face of the multiobjective sinusoidal algorithm (MOSCA), the objective function is optimized [1]. The indexes of the optimized objective function are data rate, signal-to-interference-noise ratio (SINR), power consumption, and energy efficiency, and then the optimized objective function is allocated to a neural network for resource allocation [2]. It expounds a new deep neural network convolution layer-variable convolution (vConv) layer, which learns the kernel length of data adaptively by its own cycle to realize the motif recognition of data sets with high throughput [3]. It proves the effectiveness of pretraining neural networks on different data sets and shows that in many practical cases, the convolution layer can be replaced by a smaller fully connected layer, and the accuracy degradation is relatively small [4]. In this paper, a machine learning (DL) method is proposed to accurately obtain the performance of components and obtain the key features of typical components, and the data set is prepared and trained based on the extracted indicators [5]. It introduces the method for fault damage. Because of its powerful feature extraction ability, this method can extract more advanced and abstract fault features from massive data. Experiments show that deep neural network has better feature learning and classification performance in the field of fault diagnosis [6]. This paper introduces the background of semantic segmentation, then divides the semantic segmentation methods based on deep learning into five categories, and presents the advantages and disadvantages of each category [7]. The surrogate model is established by using polynomial chaotic expansion and a deep neural network, and the Sobol exponent needed to identify the influence of soil parameters on dam behavior is calculated [8]. In this paper, a new pruning-based DNNs model is proposed without affecting the expression ability of DNNs. By mining more compact structures and learning the effective weights, the computational overhead of DNNs is reduced [9]. We replace these empirical parameters with dynamic values of machine learning, which perfectly improves the accuracy of the extended model and uses complex neural networks to generate dynamic values to reproduce orbital energy and density based on density functional theory [10]. In this paper, the prediction ability of deep neural network relative to other machine learning technologies is established, and the future range of deep learning in multiparameter time series prediction is shown [11]. Our results are helpful to improve the estimation performance of structural variables of Arctic forests by using the concepts of image sampling and input features proposed in this paper [12]. This method also has the advantage of transfer learning; that is, DNN trained on one battery data set can use less training data to improve the curve estimation of other batteries running in different scenarios [13]. It will make a simple scientific overview of machine learning [14]. Learning-based computer generated holography (CGH) provides a fast way to generate holograms for holographic displays [15]. In REPAID, the multifocus image is first reconstructed from a single all-optical image and then up-sampled by a specially designed depth neural network suitable for real scenes, and finally, a full-focus image with the high spatial and temporal resolution is generated [16]. It introduces a regularized chain of deep and complex brain structure networks to handle classification tasks from multiple annotators [17]. A complex brain structure network for linear B cell epitope prediction is introduced [18]. The most relevant deep learning-based methods and the most advanced graphic page object detection in document images are discussed [19]. Materials informatics is an emerging field, which allows us to predict the properties of materials and has been applied to various research and development fields, such as materials science [20]. A new two-layer depth neural network structure is proposed, which can reconstruct the self-organized humanlike deformation shape from the depth framework by combining the inherent parameters of the camera [21]. Deep neural network for privacy protection becomes essential because of the need to maintain personal privacy and confidentiality of sensitive data and has attracted the attention of many researchers. With the wide application of neural network as a service in the unsecured cloud environment, the importance of privacy protection network is increasing day by day [22]. CNN is a new image recognition technology. Compared with the standard manual feature extraction method, it does not need explicit feature engineering and extraction and produces efficient results [23]. Its performance is satisfactory in both novelty detection and fault diagnosis, which is superior to other advanced methods. This study proposes a new fault diagnosis method, which can not only diagnose defects of known types but also detect defects of unknown types [24]. We propose a new algorithm called depth feature selection, which is used to estimate sparse parameters and other parameters at the same time [25].

## 2. Concept and Characteristics of Complex Network Method of Brain Structure

### 2.1. The Concept of Artificial Neural Networks

Human brain is the most complex, perfect, and effective information processing system known by human beings in exploring unknown fields. It is the advanced product of biological evolution and the cornerstone of advanced spiritual movements such as language, thinking, and emotion of the human brain. At present, human beings have little knowledge of this field. For a long time, scholars have been studying neural networks through the analysis of a series of disciplines such as neurology, psychology, cognition, mathematics, electronics, and computational science and want to dissect the structure of the human brain and its massive information processing modes. Using the complex network structure characteristics of the brain, an intelligent system similar to the human brain, which can perform some functions, is designed to deal with massive information and solve the complex problem of blending different data. It is the core goal of the development of science and technology to replace part of the labor of the human brain with a machine structure composed of electronic parts. Computer is an information processing system which uses some electronic components to carry out some memory, calculation, and information processing functions of the human brain. The speed of every electronic component in modern computers is as high as nanoseconds, while the reflection time of every nerve cell in the human brain is only millisecond units. Therefore, the complex neural network method of the brain is only a neural network which can complete some ideal function after artificial construction and processing on the basis of cognitive understanding of the brain neural network. It is a mathematical network method close to the idealized human brain nerve structure, and it is also a data processing structure based on imitating the complex nervous system structure and function of the brain. In fact, it is a complex network structure constructed by a large number of simple components interconnected with each other, which has complex nonlinearity and can carry out complex logic operations and realize some curvilinear relationships.

### 2.2. Features of Artificial Neural Networks

Although an artificial neural network is an idealized network structure based on brain structure, the complex network structure of the brain is different from the current computer and artificial intelligence structure. It has many similarities with human intelligence: the performance of a single neural unit is relatively weak, but a large number of neurons converge into a network structure, which will have interoperability and parallel processing functions and is very powerful. It has the following characteristics:

1. *Inherent Parallel Structure and Parallel Processing*. The similarity between an artificial neural network and the human brain in structure is parallel and interoperable, and the processing sequence is also parallel and simultaneous. Neurons in each layer process data at the same time. That is to say, the function of neural network processing data can be distributed and can be carried out simultaneously on multiple processing units.
2. *The Storage of Knowledge*. In the complex neural network model of brain structure, knowledge is not only stored in a fixed storage unit but all the memorized information is stored in the weights of interconnection between neurons. The content of stored information can not be seen from the weights of a single neuron, because the knowledge is stored in a distributed way.
3. *Strong Fault Tolerance*. The automatic death of brain cells every day will not affect our memory ability and thinking ability. Similarly, artificial neural networks have strong error tolerance; that is, local or partial neuron damage will not affect the subsequent and even global activity changes.
4. *Self-Adaptability*. Artificial neural network can acquire various abilities through learning. Input the input and ideal output modes into complex networks; the network adjusts the connection weights between neurons in each layer of the system according to the basic information extracted from the samples given by the original learning algorithm and stores these basic information in a specific system in the form of connection methods between neurons until the network reaches a stable state.

## 3. Sports Competition Prediction Algorithm

### 3.1. Traditional Statistical Methods

#### 3.1.1. Determining the Time Series Prediction Model

(1) *No Change Method*. The premise of this method is that the sports competition results in *T* + 1 period are the same as those in *T* period. Because this model is too idealistic, it may produce a higher prediction value for Chinese sports competition results with an obvious improvement trend.
(2) *Proportional Change Method*. It is considered that the results of sports competitions change by a certain percentage with time. The expression is as follows:
  (1) $$\begin{array}{c} C_{t+1}^{A}=C_{t}\left(1+\frac{C_{t}-C_{t-1}}{C_{t-1}}\right). \end{array}$$
(3) *Moving Average Model Method*. That is, the average of observed values in the past several periods is used as the predicted value of the prediction period. The expression is as follows:
  (2) $$\begin{array}{c} C_{t+1}^{A}=\frac{C_{t}+C_{t-1}+\cdots +C_{t-n+1}}{N}\left(t\geq N\right). \end{array}$$
(4) *Weighted Moving Average Model*. Different weights are given according to the time when each data are away from the prediction period. It is generally believed that the closer the time is away from the prediction period, the greater the weight should be given because the recent prediction value has stronger prediction ability and accuracy. The expression is as follows:
  (3) $$\begin{array}{c} C_{t+1}^{A}=\frac{a_{0}C_{t}+a_{1}C_{t-1}+\cdots +a_{N-1}C_{t-n+1}}{N}\left(t\geq N\right). \end{array}$$
(5) *Exponential smoothing model*.

It is a special weighted average method, which uses the weighted average of the previous observation value and the predicted value as the predicted value of the next period. The calculation formula is

(4) $$\begin{array}{c} C_{t+1}^{A}=aC_{t}+\left(1-a\right)C_{t}^{A}. \end{array}$$

#### 3.1.2. Stochastic Time Series Model

This is the most commonly used method in the time sequential model method to find out the trend of sports competition results with a certain factor by regression method. The independent variable in the model can be any one of the influencing factors of sports competition results. People usually use time as the independent variable.

### 3.2. Evaluation Criteria of Sports Competition

The parameters of absolute average error, correlation coefficient, and reliability of output data are used to evaluate the model. Because they are neither affected by the size of the sample nor by the restriction of sample units on other different models, the comparability is strong. They can be obtained by the following formula.

Absolute mean difference:

(5) $$\begin{array}{c} \text{MAPE}=\frac{\sum_{i=1}^{n}\left|x_{i}-y_{i}\right|/y_{i}}{n}\times 100\%. \end{array}$$

Correlation coefficient:

(6) $$\begin{array}{c} R=\frac{\sum_{i=1}^{m}\left(x_{i}\times y_{i}\right)}{\sqrt{\sum_{i=1}^{m}{\left(x_{i}\right)}^{2}\times \sum_{i=1}^{m}{\left(y_{i}\right)}^{2}}}\;. \end{array}$$

Data reliability:

(7) $$\begin{array}{c} Z=\frac{\sum_{i=1}^{n}j}{n}\times 100\%, \end{array}$$

where |*x*_*i*_ − *y*_*i*_|/*y*_*i*_ ≤ 0.15,  *j*=1, otherwise *j* = 0.

### 3.3. Artificial Neural Network Algorithm

The expression of the input sum of neurons is as follows:

(8) $$\begin{array}{c} \text{net}=\sum_{i=1}^{n}P_{i}W_{i}.\; \end{array}$$

Neuron output:

(9) $$\begin{array}{c} o=f\left(\text{net}-\theta \right). \end{array}$$

Artificial neural network learning is carried out under the condition that the input mode and ideal output mode are known. Its comprehensive error often adopts the sum of squares of errors.

(10) $$\begin{array}{c} E_{k}=\frac{1}{2}\sum_{j}^{Q}{\left(O_{j}^{k}-o_{kj}\right)}^{2}. \end{array}$$

#### 3.3.1. BP Neural Network

(1) Calculate the output values of hidden layer and output layer nodes:
Hidden node output:
  (11) $$\begin{array}{c} y_{i}=f\left(\underset{j}{\sum }w_{xj}x_{j}-\theta_{i}\right). \end{array}$$
Output node:
  (12) $$\begin{array}{c} o_{i}=f\left(\underset{j}{\sum }T_{ij}y_{i}-\theta_{i}\right). \end{array}$$
(2) Calculate the error of output layer and hidden layer:
All sample errors:
  (13) $$\begin{array}{c} E=\sum_{k=1}^{p}e_{k}\leq \varepsilon . \end{array}$$
One sample error:
  (14) $$\begin{array}{c} e_{k}=\overset{n}{\underset{i=1}{\sum }}\left|t_{i}^{\left(k\right)}-o_{i}^{\left(k\right)}\right|. \end{array}$$
Output layer node error:
  (15) $$\begin{array}{c} \delta_{i}=\left(t_{i}-o_{i}\right)\cdot o_{i}\cdot \left(1-o_{i}\right). \end{array}$$
Hidden layer node error:
  (16) $$\begin{array}{c} \delta_{i}=y_{i}\left(1-y_{i}\right)\sum_{i}\delta_{i}T_{ij}. \end{array}$$
(3) Correct the node weights and closed values of output layer and hidden layer:

Output layer node weight correction:

(17) $$\begin{array}{c} T_{ij}\left(k+1\right)=T_{ij}\left(k\right)+\eta \delta_{i}y_{i}. \end{array}$$

Output layer node threshold correction:

(18) $$\begin{array}{c} \theta_{i}\left(k+1\right)=\theta_{i}\left(k\right)+\eta \delta_{i}. \end{array}$$

Modification of node weight value in hidden layer:

(19) $$\begin{array}{c} W_{ij}\left(k+1\right)=W_{ij}\left(k\right)+\eta \delta_{i}x_{i}. \end{array}$$

Hidden layer node threshold correction:

(20) $$\begin{array}{c} \theta_{i}\left(k+1\right)=\theta_{i}\left(k\right)+\eta \delta_{j}. \end{array}$$

#### 3.3.2. BP Algorithm Improvement

*(1) Additional Momentum Value Method*. The method is based on the back propagation method, adding a dynamic change quantity proportional to the last weight and threshold value to each change of weight and threshold value and generating new changes of weight and threshold value according to the back propagation method. The adjustment formula of weight and threshold value with additional momentum factor is as follows:

(21) $$\begin{array}{c} \Delta X\left(k+1\right)=mc\times \Delta X\left(k\right)+lr\times \frac{\partial E}{\partial X}\;\left(0<lr\leq 1\right). \end{array}$$

*(2) Adaptive Learning Rate Method*. Adjusting the learning rate based on self-adaptation rules is beneficial to shorten the time. The learning rate is too small and the convergence rate is too slow; if the range of learning rate selection is too large, it may change too much, resulting in data dispersion. Therefore, a unique improved algorithm suitable for adaptive adjustment appears, and its weight and gap value update expression is

(22) $$\begin{array}{c} \Delta X=lr\times \frac{\partial E}{\partial X},\\ \Delta X\left(k+1\right)=mc\times \Delta X\left(k\right)+lr\times mc\times \frac{\partial E}{\partial X}. \end{array}$$

Adjustment formula of adaptive learning rate *lr* is as follows:

(23) $$\begin{array}{c} lr\left(k+1\right)=\left\{\begin{array}{c} 1.06lr\left(k\right)mse\left(k+1\right)<mse\left(k\right),\\ 0.6lr\left(k\right)mse\left(k+1\right)>1.05mse\left(k\right),\\ lr\left(k\right). \end{array}\right. \end{array}$$

*(3) Levenberg-Marquardt Optimization Method*. The weight and threshold update formula is

(24) $$\begin{array}{c} X_{k+1}=X_{k}-{\left(J^{T}J+\mu I\right)}^{-1}J^{T}e. \end{array}$$

## 4. Experiment

### 4.1. Simulation Experiment

**Figure 1:**
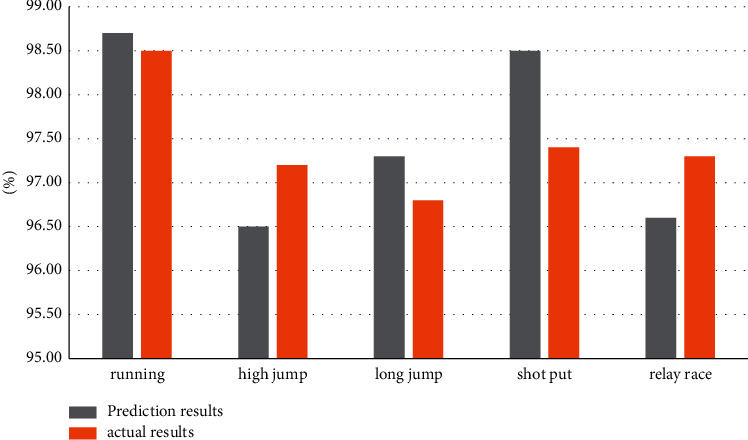
Comparison between predicted results and actual values.

In order to effectively predict sports performance, 500 data samples of sports performance in a university were collected, of which 400 were used as training samples and 100 as test samples. 400 training samples are trained by a support vector machine algorithm, and the sports scores of 100 test samples are predicted by an online computing platform. The prediction results and prediction error curves obtained by the online computing platform are shown in Table 1 and Figure 1.

Through the above-given experimental results, we can see that using this model to test the sports performance prediction results are very ideal, the experimental results show that using this model can effectively predict sports performance.

The predicted results and actual results of the above-mentioned sports events are counted into a bar chart as shown in Figure 1.

**Figure 2:**
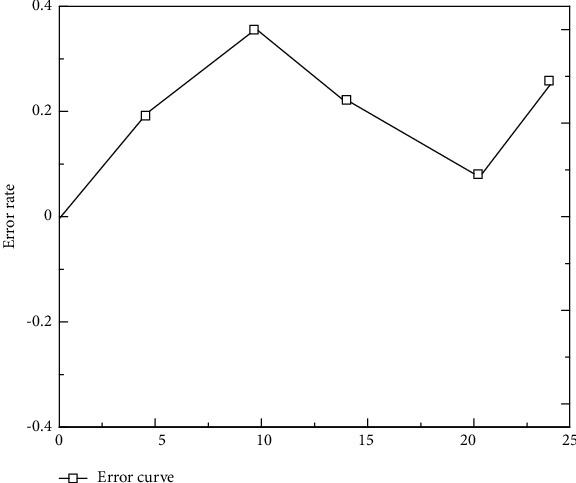
Error curve of sports events.

Five groups of sports data are selected from the training sample data set to test and verify whether the improved BP neural network has good training. The error curve after inspection is shown in Figure 2.

### 4.2. Model Comparison

The error of predicting sports results by three models is compared, and the comparative data are shown in Table 2.

According to the experimental data in Table 2, we can get that the improved neural network model with complex brain structure has higher accuracy in predicting sports events. Is the prediction result of this model equally accurate for different types of sports events? We further compare and analyze the four kinds of sports: running, ball games, long jump, and improvement. For example, the experimental data are as follows.

Select the results of four kinds of sports events in a high school sports meeting to test the universality of the model.

The model performance indicators for running sports competitions are shown in Table 3.

**Figure 3:**
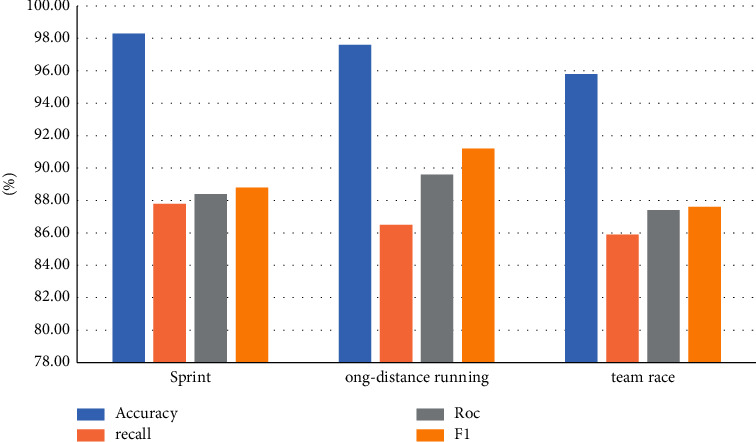
Comparison chart of running experimental data.

The model index comparison data of the above-given experimental results are counted into a bar chart as shown in Figure 3.

Model performance indicators for ball games are compared and are shown in Table 4.

**Figure 4:**
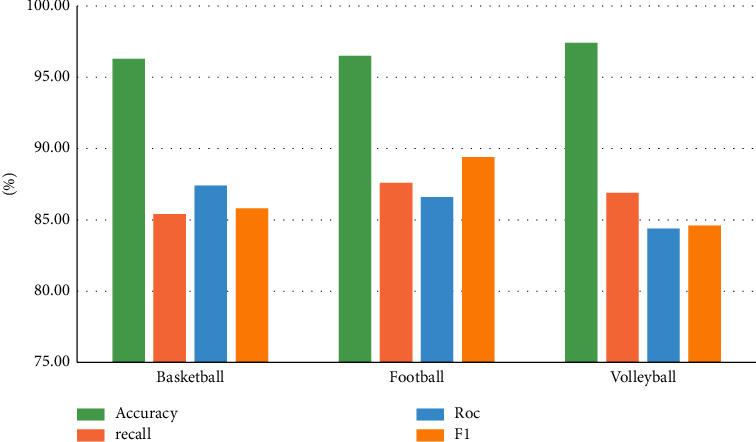
Comparison chart of ball experiment data.

The model index comparison data of the above experimental results are counted into a bar chart as shown in Figure 4.

The model performance indicators for long jump sports are shown in Table 5.

**Figure 5:**
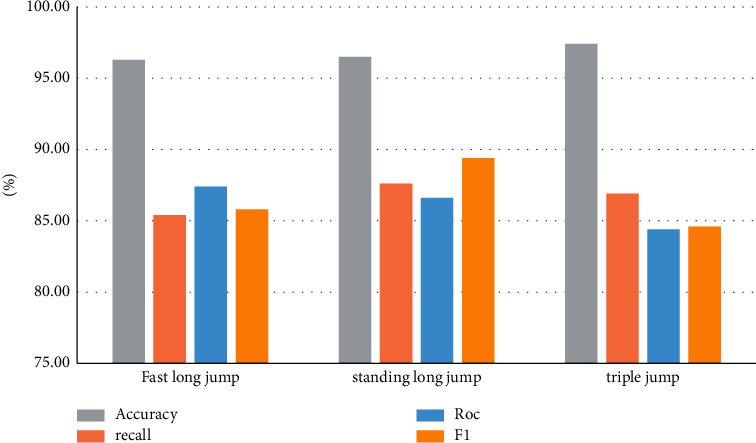
Comparison of experimental data of long jump.

The model index comparison data of the above experimental results are counted into a bar chart as shown in Figure 5.

Model performance indicators for high jump sports are shown in Table 6.

**Figure 6:**
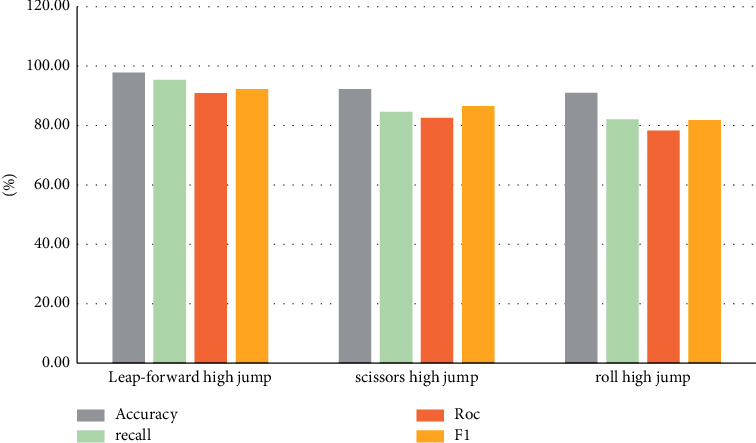
Comparison of experimental data of high jump.

The model index comparison data of the above experimental results are counted into a bar chart as shown in Figure 6.

### 4.3. Contrast Experiment

Through the previous results, it is proved that applying the single prediction of various sports events, the prediction results superior to each single item can be combined, which can further effectively improve the prediction accuracy. Therefore, this study continues to use the improved neural network model construction method of complex brain structure to establish a prediction model for all the data from 2018 to 2021 and predict the comprehensive evaluation index values in these years. The prediction results are shown in Table 7.

## 5. Conclusion

Through the preliminary establishment of the autoregressive summation model prediction model of sports competition development in China, the neural network prediction model of complex brain structure, and the improved network prediction model, it is concluded that the improved complex brain structure model prediction model has a remarkable effect, and its research results are as follows:

1. In both the neural network model and traditional regression model, a neural network with a complex brain structure is more inclined to predict the results of sports competitions
2. In the model comparison experiment, we predict the individual items of various sports events, and the prediction effect of the improved prediction model is inconsistent
3. Among the four kinds of sports in the article, the prediction effect of running is the most obvious, while the prediction effect of the other three kinds is poor
4. The prediction error of the improved neural network model is reduced by about twice as much as that of the original model

## Figures and Tables

**Table 1:** Comparison of forecast data of online computing platform.

Sports events	Prediction result (%)	Prediction error (%)	Actual result (%)
Running	98.7	0.21	98.5
High jump	96.5	0.38	97.2
Long jump	97.3	0.22	96.8
Shot put	98.5	0.13	97.4
Relay race	96.6	0.26	97.3

**Table 2:** Error comparison of each prediction model.

Model name	RMSE	MAPE
Autoregressive summation	0.321	0.531
BP neural network	0.217	0.328
Improved BP network	0.142	0.249

**Table 3:** Comparison of performance indexes of model indexes in running sports.

Sports events	Accuracy (%)	Recall (%)	ROC (%)	F1 (%)
Sprints	98.3	87.8	88.4	88.8
Long-distance races	97.6	86.5	89.6	91.2
Team races	95.8	85.9	87.4	87.6

**Table 4:** Comparison of performance indexes of model indexes in ball games.

Sports events	Accuracy (%)	Recall (%)	Roc (%)	F1 (%)
Basketball	96.3	85.4	87.4	85.8
Football	96.5	87.6	86.6	89.4
Volleyball	97.4	86.9	84.4	84.6

**Table 5:** Comparison of performance indexes of model indexes in long jump sports events.

Sports events	Accuracy (%)	Recall (%)	Roc (%)	F1 (%)
Fast walk long jump	95.3	85.4	80.4	82.3
Standing long jump	98.5	90.6	92.6	92.3
Triple jump	97.6	86.7	82.4	85.8

**Table 6:** Comparison of performance indexes of model indexes in high jump sports events.

Sports events	Accuracy (%)	Recall (%)	Roc (%)	F1 (%)
Leap-forward	97.8	95.4	90.9	92.3
Scissors high jump	92.3	84.6	82.6	86.5
Boiling high jump	91.0	82.1	78.3	81.8

**Table 7:** Comparison of prediction accuracy of each model.

Year	Absolute value of prediction error of autoregressive sum model	Absolute value of prediction error of BP neural network model	Absolute value of prediction error of improved BP model
2018	0.8321	1.2093	0.6512
2019	0.9682	0.9827	0.4381
2020	0.7765	0.8821	0.3165
2021	0.5932	0.5411	0.2109
Errors	3.17	3.6152	1.6167

## Data Availability

The experimental data used to support the findings of this study are available from the corresponding author upon request.

## References

[1] Jaseena K. U., Kovoor Binsu C. (2021). An improved multivariate weather prediction model using deep neural networks and particle swarm optimisation. *Journal of Information and Knowledge Management*.

[2] Purushothaman K. E., Nagarajan V. (2021). Evolutionary multi-objective optimization algorithm for resource allocation using deep neural network in 5G multi-user massive MIMO. *International Journal of Electronics*.

[3] Li J. Y., Jin S., Tu X. M., Ding Y., Gao G. (2021). Identifying complex motifs in massive omics data with a variable-convolutional layer in deep neural network. *Briefings in Bioinformatics*.

[4] Gusak J., Daulbaev T., Ponomarev E., Cichocki A., Oseledets I. (2021). Reduced-order modeling of deep neural networks. *Computational Mathematics and Mathematical Physics*.

[5] Wang P. (2021). Fast and accurate performance prediction and optimization of thermoelectric generators with deep neural networks. *Advanced Materials Technologies*.

[6] Tong S. (2021). Application of deep neural network in fault diagnosis. *International Core Journal of Engineering*.

[7] Zhou W., Chen K. (2021). A brief survey on semantic segmentation based on deep neural network. *International Core Journal of Engineering*.

[8] Shahzadi G., Soulaïmani A. (2021). Deep neural network and polynomial chaos expansion-based surrogate models for sensitivity and uncertainty propagation: an application to a rockfill dam. *Water*.

[9] Chen Q. (2021). An efficient pruning scheme of deep neural networks for internet of things applications. *EURASIP Journal on Applied Signal Processing*.

[10] Zubatiuk T., Nebgen B., Lubbers N. (2021). Machine learned Hückel theory: interfacing physics and deep neural networks. *The Journal of Chemical Physics*.

[11] Srivastava P. R., Prajwal E., Zuopeng J. Z. (2021). Deep neural network and time series approach for finance systems. *Journal of Organizational and End User Computing*.

[12] Heikki A. (2021). Deep neural networks with transfer learning for forest variable estimation using sentinel-2 imagery in boreal forest. *Remote Sensing*.

[13] Tian J., Xiong R., Shen W., Lu J., Yang X. G. (2021). Deep neural network battery charging curve prediction using 30 points collected in 10 min. *Joule*.

[14] Mukhamediev R. I., Symagulov A., Kuchin Y., Yakunin K., Yelis M. (2021). From classical machine learning to deep neural networks: a simplified scientometric review. *Applied Sciences*.

[15] Wu J., Liu K., Sui X., Cao L. (2021). High-speed computer-generated holography using an autoencoder-based deep neural network. *Optics Letters*.

[16] Yu M., Gu Y., Jiang Z. (2021). REPAID: resolution-enhanced plenoptic all-in-focus imaging using deep neural networks. *Optics Letters*.

[17] Julián G. G. (2021). Regularized chained deep neural network classifier for multiple annotators. *Applied Sciences*.

[18] Collatz M., Mock F., Barth E., Holzer M., Sachse K., Marz M. (2021). EpiDope: a deep neural network for linear B-cell epitope prediction. *Bioinformatics*.

[19] Bhatt J., Hashmi K. A., Afzal M. Z., Stricker D. (2021). A survey of graphical page object detection with deep neural networks. *Applied Sciences*.

[20] Atsushi K., Toshifumi K., Kikuchi J. (2021). Solubility prediction from molecular properties and analytical data using an in-phase deep neural network (Ip-DNN). *ACS Omega*.

[21] Audrius K. (2021). HUMANNET—a two-tiered deep neural network architecture for self-occluding humanoid pose reconstruction. *Sensors*.

[22] Raghida E. S., Sedgh Gooya E., Alfalou A., Khalil M. (2021). Privacy-preserving deep neural network methods: computational and perceptual methods—an overview. *Electronics*.

[23] Mushtaq F., Misgar M. M., Kumar M., Khurana S. S. (2021). UrduDeepNet: offline handwritten Urdu character recognition using deep neural network. *Neural Computing & Applications*.

[24] Yang Z., Gjorgjevikj D., Long J., Zi Y., Zhang S., Li C. (2021). Sparse autoencoder-based multi-head deep neural networks for machinery fault diagnostics with detection of novelties. *Chinese Journal of Mechanical Engineering*.

[25] Yao C. (2021). Nonlinear variable selection via deep neural networks. *Journal of Computational & Graphical Statistics*.

